# RNAi Mediated Gene Silencing of Detoxification Related Genes in the *Ectropis oblique*

**DOI:** 10.3390/genes13071141

**Published:** 2022-06-24

**Authors:** Cui Peng, Heng Yin, Yang Liu, Xin-Fang Mao, Zhong-Yuan Liu

**Affiliations:** College of Chemical Engineering, Sichuan University of Science and Engineering, 180 Xueyuan Street, Zigong 643000, China; 320086001207@stu.suse.edu.cn (C.P.); 3190817z202@stu.suse.edu.cn (H.Y.); 320086001201@stu.suse.edu.cn (Y.L.); maoxinfang@suse.edu.cn (X.-F.M.)

**Keywords:** *Ectropis oblique*, RNA interference, QRT-PCR, pesticide, detoxification metabolism

## Abstract

*Ectropis oblique* is one of the main pests that feed on tea leaves. At present, the main control method is chemical control, but the long-term use of insecticides has been related to the development of insect resistance. One of the resistance mechanisms is the upregulation of relevant detoxification enzymes for defense. In this study, four genes with increased expression were screened from the gene sequences annotated from the transcriptome data of deltamethrin-treated larvae of *E*. *oblique*, which are acid phosphatase *EoACP138*, and cytochrome P450 *EoCYP316*, carboxylesterase *EoCarE592* and acetylcholine esterase *EoAchE989*, respectively. The fourth instar larvae of *E*. *oblique* were stimulated by deltamethrin, chlorpyrifos and fenpropathrin respectively, and the expression levels of the genes were detected by qRT-PCR. The result showed that all four genes’ expression had significantly increased under the stimulation of three insecticides. RNAi technology was used to silence the expression of genes of *EoACP138*, *EoCYP316*, *EoCarE592* and *EoAchE989* in the fourth instar larvae of *E. oblique*. The change in the expression levels of the above genes in the larvae treated with dsRNA and stimulated with pesticides was determined by qRT-PCR. The target genes have been effectively silenced after feeding on dsRNA and higher sensitivity with higher mortality to pesticides was observed in the larvae interfered with dsRNA. The above genes are related to the detoxification and metabolism of resistance of *E. oblique*, which lays a foundation for further study on the mechanism of insecticide resistance in *E. oblique*.

## 1. Introduction

The tea plant is a popular cash crop that has been grown in more than 60 countries and consumed in more than 100 countries [1]. The leaves are processed into tea and become one of the popular non-alcoholic beverages [2,3]. Tea is beneficial to people’s health owing to its bioactive components including catechins, polyphenols, theanine, caffeine, volatile oil, flavonoids, vitamins and medicinal properties [4,5,6]. *E. oblique* (Lepidoptera: Geometridae) is one of the most destructive chewing pests of tea trees. In the early stage, the larvae feed on tender leaves and shoots. With a rapid growth, the larvae of the fourth and 5th instars bite the leaves to form notches, which will seriously affect the yield and quality of tea [7,8,9]. There are various control methods for the pest, including manpower, entrapment, a chemical pesticide, microorganism pesticide, biological guided missiles and agriculture expert systems; however, chemical pesticides are the main control measures. The excessive use of chemical pesticides will lead to environmental pollution, the generation of insecticide resistance, and the residual pesticides would endanger human health [10,11,12,13,14]. For these reasons, more innovative and safe methods are needed for the control of *E. oblique* and other pests.

At present, RNA interference (RNAi) is mainly used to study the function of genes; in insects, this technology has become a research hotspot and has been applied to pest control [15,16,17,18]. RNAi is a gene-silencing technique using double-stranded RNA (dsRNA). DsRNA is mainly introduced into insects by oral administration, immersion or injection [19], digested into siRNA (short interfering RNA) by endonuclease, and finally leads to the specific degradation of messenger RNA through the action of RNA-induced silencing complex (RISC). Since the discovery that double-stranded RNA can mediate specific interference in *Caenorhabditis elegans* [20], RNAi technology has been successfully applied to the research of many insects. After being injected with dsRNA, the expression of marker genes of the pea aphid *Acyrthosiphon pisum* was inhibited by about 40% [21]. Jing Lü et al., proved that the survival rate of *Henosepilachna vigintioctopunctata* was significantly reduced with the target gene effectively silenced after feeding on dsRNA targeting vATPase-B [22].

Many studies have found the resistance mechanisms to insecticide was highly related to the upregulation of detoxifying enzyme genes [23]. In many cases, insects can respond to the stimulation of pesticides through the detoxification metabolic pathway. The metabolic enzymes related to detoxification mainly include esterase (EST), cyto-chrome P450 enzyme family (cytochrome P450, CYP) and glutathione S-transferase (GST) [24,25,26,27]; however, the resistance mechanisms of different pests and the related genes which responded to different pesticides are different. Therefore, RNAi technology can be used to silence the relevant detoxification gene base in insects in order to study its function in detecting the sensitivity of insects to pesticides and their mortality rate.

In this study, four genes *EoACP138*, *EoCYP316*, *EoCarE592* and *EoAchE989* from *E*. *oblique* were selected based on the previous studies of transcriptome [28], and the relationship between the genes and pesticide resistance of *E. oblique* has been further studied by RNAi technology. The results showed that the silencing rate of the four genes was higher than 70%, and the sensitivity of *E*. *oblique* to pesticides was improved; it paves the way for potential application in pest control and the development of detoxification drugs in the future.

## 2. Materials and Methods

### 2.1. Insect Rearing

*E. oblique* used in this research was provided by the Tea Research Institute of the Chinese Academy of Agricultural Sciences (Hangzhou, Zhejiang, China), and were reared and propagated with fresh tea leaves in groups by our laboratory. Fresh tea leaves were picked from Jianshan Scenic Spot in Zigong, Sichuan province. The feeding conditions were as follows: temperature 22 ± 2 °C, photoperiod 14:10 (light: dark), relative humidity 65 ± 5%.

### 2.2. Total RNA Extraction and cDNA Synthesis

The total RNA of larvae was extracted using TriZol Reagent (Sangon Biotech, Shanghai, China) following the manufacturer’s manual. Total RNA was treated with DNase I after the extraction procedure to remove genome DNA from the samples. The concentration and purity of 1 μL RNA solution were measured by Nanodrop, and the quality of RNA was further determined by 1.5% agarose gel electrophoresis. First-strand cDNA was synthesized with 2 μL total RNA as a template using M-MuLV First Strand cDNA Synthesis Kit (Sangon Biotech, Shanghai, China). The reaction conditions of PCR were as follows: 42 °C for 30–60 min, then the reaction was terminated at 70 °C for 10 min. The obtained cDNA can be stored at −20 °C for standby.

### 2.3. Quantitative Real-Time PCR Analysis

Based on the base sequences of four genes, *EoACP138* (GenBank accession number ON110137), *EoCYP316* (GenBank accession number ON110138), *EoCarE592* (GenBank accession number ON110139) and *EoAchE989* (GenBank accession number ON110140), and the design principles of the qRT-PCR primers, Primer premier 5 was used to design the corresponding primers, while a fragment of *E. oblique* 18S rRNA was used as an internal reference for qRT-PCR. The primers for each gene are listed in Table 1.

#### 2.3.1. Treatment of *E. oblique* with Deltamethrin

In the preliminary work of the laboratory, the pesticides were diluted to different concentrations, and the fresh tea leaves were immersed in the diluent for 10 s, taken out, and dried in air. The control group was immersed in deionized water. Then, the starved larvae were fed for 48 h, and the mortality rate was recorded. The corresponding LC50 value was calculated based on the deaths at different concentrations. Where the LC50 of deltamethrin was 25 μg/mL, it was diluted with water to 25 μg/mL, and the picked intact leaves (oval) with similar leaf age were completely immersed in the deltamethrin diluent for 10 s, and taken out and naturally air-dried. The fourth instar larvae were selected and divided into 4 groups (10 larvae per group). The larvae were fed for 0 h, 12 h, 24 h, and 48 h and frozen in liquid nitrogen for RNA extraction. Three biological replicates and three technical replicates were set. The transcription levels of *EoCYP316*, *EoACP138*, *EoCarE592* and *EoAchE989* in *E. oblique* larvae at different time points after deltamethrin exposure were detected by quantitative real-time PCR. The qRT-PCR reaction system includes 2 × transstart tip green qPCR Supermix 10 μL, the forward and reverse primers (10 μM/L) each 0.4 μL, cDNA template 1 μL, Nuclease-free Water 8.2 μL. QRT-PCR conditions were: 95 °C for 2 min; followed by 40 cycles of 95 °C for 10 s, and 60 °C for 20–30 s. 2^−∆∆CT^ method was used to calculate the relative expression of genes. 

#### 2.3.2. Fenpropathrin and Chlorpyrifos Treatment for *E. oblique*

Fenpropathrin was diluted to LC50 (0.861 mg/mL) and chlorpyrifos to LC50 (0.725 mg/mL) based on preliminary laboratory calculations. Tea leaves with similar leaf ages were completely immersed in the dilution of fenpropathrin and chlorpyrifos respectively for 10 s and removed for air drying. Fourth instar larvae were selected and divided into 4 groups (10 larvae per group) and fed on leaves treated with the diluted fenpropathrin and chlorpyrifos respectively. The larvae were treated with liquid nitrogen at 0 h and 24 h after feeding for RNA extraction (10 larvae per group). Three biological replicates and three technical replicates were set. The transcription levels of *EoCYP316*, *EoACP138*, *EoCarE592* and *EoAchE989* in the larvae were detected by qRT-PCR. The relative expression of genes was calculated using the 2^−∆∆CT^ method. 

### 2.4. dsRNA Synthesis

Based on the base sequences of the four genes, the primers were designed including the T7 promoter (TAATACGACTCACTATAGGG) and GATCAC promotor group. The green fluorescence protein gene (*GFP*) was used as a control gene and the primers were designed as listed in Table 2. A large amount of the purified DNA template was obtained by PCR using the plasmid DNA containing the target gene as the template. Double-strand RNA was synthesized according to the In Vitro Transcription T7 Kit specification from Japan’s TaKaRa Company. The dsRNA was purified by using Easy Pure^®^ RNA Purification Kit from Beijing Quan-Shi Jin Company, and dsRNA homogeneity and concentration were determined by agarose gel electrophoresis and Nanodrop.

### 2.5. RNA Interference

The dsRNA was delivered to *E. oblique* larvae by injection. The purified *dsGFP*, *dsEoCYP316*, *dsEoACP138*, *dsEoCarE592* and *dsEoAchE989* were diluted with RNase-free water to 200 ng/μL, 100 ng/μL, 50 ng/μL and 25 ng/μL, respectively. The larvae of the fourth instar with appropriate size were selected and divided into eight groups, six for each group. Each larva was injected with 2 μL dsRNA diluent to yield 50 ng, 100 ng, 200 ng, and 400 ng. After the injection, the larvae were fed with tea leaves for 1 day. Then, the larvae were rapidly frozen with liquid nitrogen for the extraction of total RNA. The change in the target gene’s expression would be detected by qRT-PCR. 

### 2.6. Effect of RNAi on the Survival of E. oblique

In order to further study whether the injection of dsRNA of different genes will affect the survival of *E. oblique*, 2 μL of dsRNA at a concentration of 100 ng/μL was injected into the abdomen of larvae. Groups treated with *dsEoACP138*, *dsEoCYP316*, *dsEoCarE592* and *dsEoAchE989* were experimental groups, and the group treated with *dsGFP* and TE buffer was used as the control group (30 larvae per group). After injection for 2 days, the physiological conditions of insects were observed, and the mortality rate was calculated. The experiment was repeated three times.

### 2.7. Expression of Detoxification Related Genes in RNAi E. oblique Stimulated by Insecticides

The fourth instar larvae were injected with 200 ng *dsGFP* or *dsEoACP138*, respectively, and fed with untreated fresh tea leaves for one day (54 larvae per group). Then, 54 larvae were injected with the same dsRNA of each group, divided into six groups, and fed with tea leaves treated with deltamethrin, fenpropathrin and chlorpyrifos (LC50), respectively, for 0 h or 24 h, respectively. After the treatment was completed, the insects were snap-frozen with liquid nitrogen. Total RNA was extracted, and cDNA was obtained by reverse transcription. Three biological replicates and three technical replicates were set. The transcript levels of *EoCYP316*, *EoACP138*, *EoCarE592* and *EoAchE989* of *E. oblique* larvae stimulated by different insecticides were detected by qRT-PCR. The reaction procedure was the same as that in the Section 2.3.1. The same process was applied to the test groups for *dsEoCYP316*, *dsEoCarE592* and *dsEoAchE989*.

### 2.8. Determination of Toxicity of Three Insecticides after RNA Interference

Larvae of the fourth instar were collected and divided into six groups (30 larvae per group), five of which were injected with 200 ng of *dsGFP*, *dsEoACP138*, *dsEoCYP316*, *dsEoCarE592* and *dsEoAchE989*, respectively. One group with no injection was used as a control. After the injection, the larvae were fed with untreated fresh tea leaves for one day. Then, each group were fed tea leaves treated with deltamethrin (17.5 μg/mL), Chlorpyrifos (0.363 mg/mL) or fenpropathrin (0.43 mg/mL) and mortality was calculated after 48 h.

### 2.9. Statistical Analysis

The relative gene expression was calculated by the 2^−ΔΔCT^ method. The expression levels of *EoACP138*, *EoCYP316*, *EoCarE592* and *EoAchE989* genes were the relative expression levels compared with the reference gene 18S rRNA. Statistical significance of results was assessed using a one-way analysis of variance followed by Tukey’s multiple comparison test or were analyzed by an independent sample *T*-test in the biostatistical software SPSS, depending on the number of experimental groups under analysis. 

## 3. Results

### 3.1. Quantitative Real-Time PCR Analysis

#### 3.1.1. Treatment of *E. oblique* with Deltamethrin

**Figure 1:**
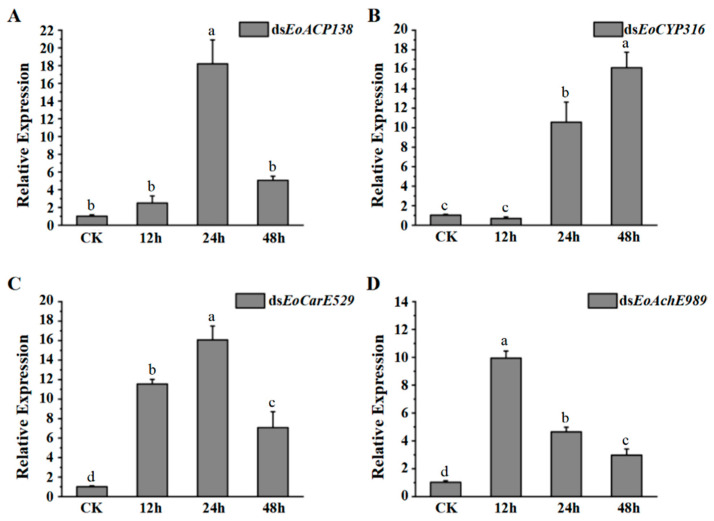
The gene expression of fourth instar larvae after deltamethrin was stimulated. (**A**) *EoACP138* (**B**) *EoCYP316* (**C**) *EoCarE592* (**D**) *EoAchE989*. Where CK was the 0 h treatment control, and 18S rRNA was used as the internal reference gene for qRT-PCR with three replicates per treatment. Each bar represents the mean ± SD. Different letters indicate significant differences (*p* < 0.05) by using the Tukey test to compare in ANOVA.

Results of quantitative real-time PCR showed that the expression level of EoACP138 (Figure 1A) and EoCYP316 (Figure 1B) in the fourth instar larvae was significantly up-regulated at 24 h under deltamethrin treatment, then, it was down-regulated at 48 h for EoACP138 while consistent up-regulation was observed for EoCYP316 at 48 h. The expression of gene EoCarE529 (Figure 1C) and EoAchE989 (Figure 1D); however, was significantly up-regulated at 12 h. Compared to the consistent down-regulation for the gene of EoAchE989 at 24 h and 48 h, it was persistently up-regulated at 24 h and down-regulated at 48 h for the gene of EoCarE529; these results indicated that the expression levels of four detoxification-related genes were significantly increased when the larvae were stimulated by the pesticide, especially after 24 h.

#### 3.1.2. Fenpropathrin and Chlorpyrifos Treatment for *E. oblique*

**Figure 2:**
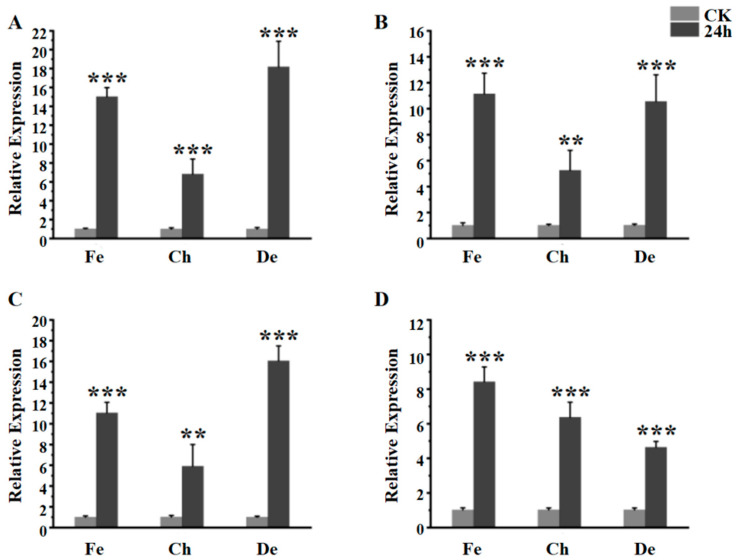
The gene expression of fourth instar larva after Chlorpyrifos, deltamethrin and fenpropathrin stimulated. (**A**) *EoACP138* gene (**B**) *EoCYP316* gene (C) *EoCarE592* gene (**D**) *EoAchE989* gene, where CK was the 0 h treatment control, Fe was fenpropathrin, Ch was chlorpyrifos, and De was deltamethrin. Each bar represents the mean ± SD. Asterisks indicate significant differences between 0 h and 24 h expression. ** = *p* < 0.01, and *** = *p* < 0.001.

The gene expression of the fourth instar larva after fenpropathrin and chlorpyrifos stimulated at 24 h was detected by qRT-PCR. Combined with the results of deltamethrin treatment, the gene expression of *EoACP138*, *EoCYP316*, *EoCarE592* and *EoAchE989* was significantly upregulated after exposure to each insecticide for 24 h compared with the control at 0 h (Figure 2A–D). The up-regulated expression levels of *EoACP138*, *EoCYP316* and *EoCarE592* for the different pesticide treatments showed the same trend (Figure 2A–C): the lower level for the chlorpyrifos treatment and higher level for fenpropathrin and deltamethrin treatment. For *EoAchE989*, the fenpropathrin treatment exhibited higher relative expression, followed by chlorpyrifos and deltamethrin (Figure 2D).

### 3.2. dsRNA Synthesis

**Figure 3:**
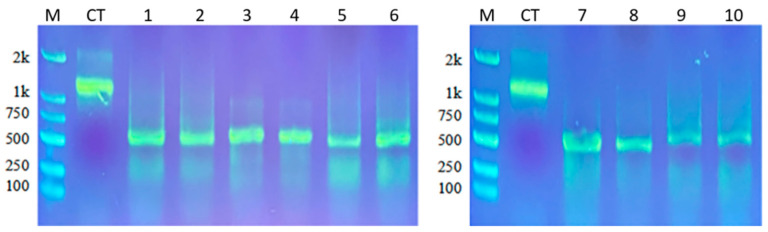
Results of dsRNA synthesis. M: Marker CT: Control Template 1–2: *EoACP138* dsRNA 3–4: *EoCYP316* dsRNA 5–6: *EoCarE592* dsRNA 7–8: *EoAchE989* dsRNA 9–10: *GFP* dsRNA.

Results of agarose gel electrophoresis showed that purified dsRNA of EoACP138, EoCYP316, EoCarE592, EoAchE989, and GFP as control synthesized in vitro had a single band in the gel, indicating the high purity of synthesized dsRNA (Figure 3). The dsRNA concentrations, measured by Nanodrop, are as follows: 365.3 ng/µL of GFP, 427.1 ng/µL of EoACP138, 378.4 ng/µL of EoCYP316, 478.2 ng/µL of EoCarE592, and 586.4 ng/µL of EoAchE989.

### 3.3. RNA Interference

**Figure 4:**
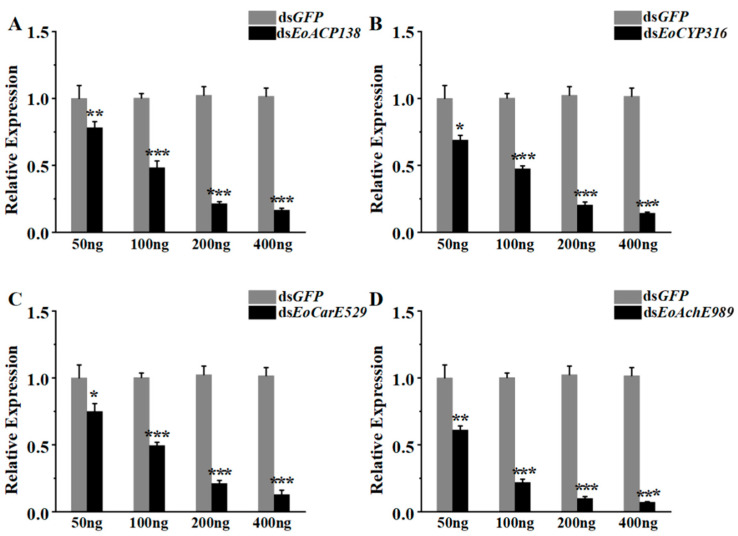
RNAi efficiency analysis of genes related to detoxification from *E. oblique* after injection of dsRNA. (**A**) The expression analysis of *EoACP138* at 24 h after injection of *dsEoACP138* by qRT-PCR. (**B**) The expression analysis of *EoCYP316* at 24 h after injection of *dsEoCYP316* by qRT-PCR. (**C**) The expression analysis of *EoCarE592* at 24 h after injection of *dsEoCarE592* by qRT-PCR. (**D**) The expression analysis of *EoAchE989* at 24 h after injection of *dsEoAchE989* by qRT-PCR. Each bar represents the mean ± SD. Asterisks indicate significant differences in expression level between *GFP* and four different genes after RNAi. * = *p* < 0.05, ** = *p* < 0.01, and *** = *p* < 0.001.

The efficiency of RNA interference depends on the concentration of dsRNA and the selection of dsRNA fragments. The fragments of dsRNA have been determined, therefore, the selection of the appropriate amount of dsRNA becomes important for the silencing of genes. As shown in Figure 4A, the gene expression levels were decreased by 22%, 52%, 79%, and 84% after injection of 50 ng, 100 ng, 200 ng, and 400 ng *dsEoACP138*, respectively. With the increasing dsRNA amount, the interference of *dsEoACP138* on the target gene became more effective. The silencing effect of *dsEoCYP316* was broadly similar to that of *dsEoACP138*, resulting in 31%, 53%, 82% and 87% gene knockdown of *EoCYP316* with the dsRNA amount increasing from 50 ng, 100 ng, 200 ng to 400 ng (Figure 4B).

The silencing effects of *dsEoCarE592* (Figure 4C) and *dsEoAchE989* (Figure 4D) for the fourth instar showed roughly the same trend. After injection doses of 50 ng, 100 ng, 200 ng, and 400 ng, 25%, 51%, 79% and 87% of gene knockdown was observed for *EoCarE592* and 39%, 78%, 92% and 94% of knockdown was observed for *EoAchE989*, respectively. At the injection dsRNA amounts of 200 ng, the suppression effect on the target genes researched 79% of *EoACP138*, 82% of *EoCYP316*, 79% of *EoCarE592,* and 92% of *EoAchE989*. Therefore, dsRNA of 200 ng was determined as the interference injection amount for the following studies.

### 3.4. Effects of RNAi on the Survival of E. oblique

**Figure 5:**
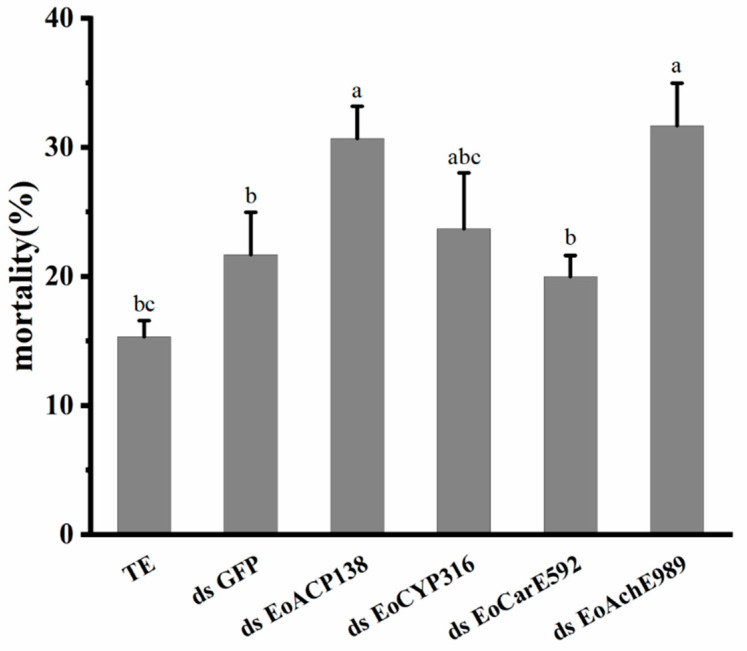
Effect of 200 ng dsRNA of four Genes on mortality of *E. oblique*. Three replicates per treatment. Each bar represents the mean ± SD. Different letters indicate significant differences (*p* < 0.05) by using a Tukey test to compare in ANOVA.

The mortality of the larvae was recorded at 48 h after injection of 200 ng of dsRNA. Compared with the mortality of 15.3% for the control group injected with TE buffer and 21.7% mortality in larvae injected with *ds GFP*, the mortality of tested groups treated with *dsEoACP138*, *dsEoCYP316*, *dsEoCarE592,* and *dsEoAchE989* were 31%, 23.7%, 19.7% and 31.7%, respectively (Figure 5). The survival of larvae interfered with dsEoACP138 and *dsEoAchE989* was significantly decreased for 48 h after injection compared to that of the larvae injected with *GFP* dsRNA.

### 3.5. Expression of Detoxification Related Genes in RNAi E. oblique Stimulated by Insecticides

**Figure 6:**
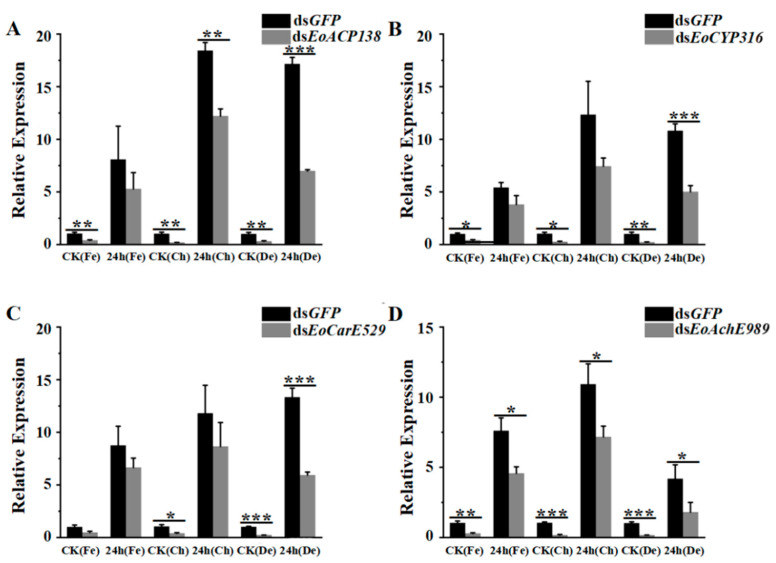
Transcriptional levels of detoxification related genes stimulated by fenprothrin, chlorpyrifos and deltamethrin respectively in *E. oblique* after RNAi. (**A**) *EoACP138*. (**B**) *EoCYP316*. (**C**) *EoCarE592*. (**D**) *EoAchE989*. Where CK was the 0 h treatment control, Fe was fenpropathrin, Ch was chlorpyrifos, and De was deltamethrin. Each bar represents the mean ± SD. * = *p* < 0.05, ** = *p* < 0.01, and *** = *p* < 0.001.

Twenty hours after injecting 200 ng of dsRNA, the expression levels of *EoACP138*, *EoCYP316*, *EoCarE592* and *EoAchE989* had been significantly decreased when compared to the control (Figure 6). The transcript levels of the detoxification-related genes of RNAi larvae stimulated by different insecticides for 24 h were detected by qRT-PCR. Although the gene expression was up-regulated after pesticide stimulation, the degree of up-regulation was significantly lower than that of the control group, which was in line with the experimental expectation. As shown in Figure 6, after treatment with chlorpyrifos, 35%, 30%, 24% and 39% of gene knockdown was observed for *EoACP138*, *EoCYP316*, *EoCarE592* and *EoAchE989* genes, respectively. After treatment with fenpropathrin, 34%, 44%, 27% and 34% of gene knockdown was observed for *EoACP138*, *EoCYP316*, *EoCarE592* and *EoAchE989* gene, respectively. After treatment with deltamethrin, 59%, 53%, 55% and 56% of gene knockdown was observed for *EoACP138*, *EoCYP316*, *EoCarE592* and *EoAchE989* gene, respectively. 

### 3.6. Determination of Toxicity of Insecticides after RNA Interference

**Figure 7:**
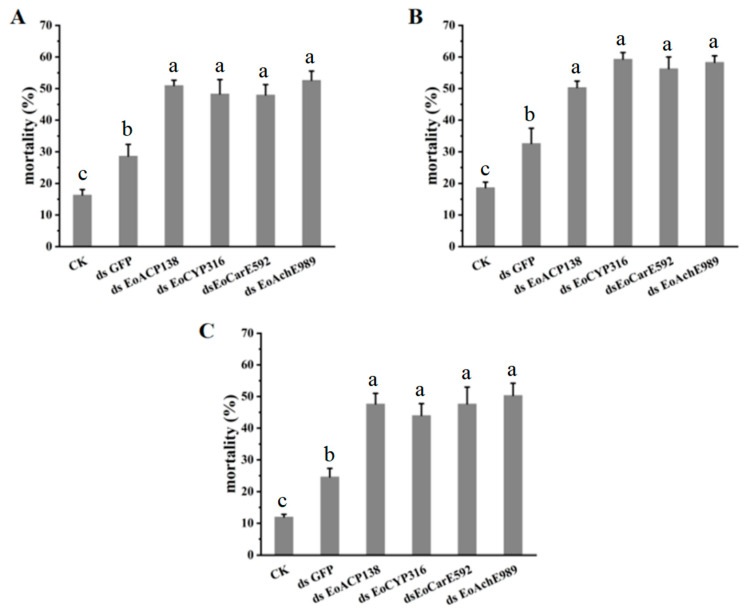
Pesticides tolerance of E. oblique after RNAi. (**A**) deltamethrin (**B**) chlorpyrifos (**C**). Fenpropathrin CK means no injection. Each bar represents the mean ± SD. Different letters indicate significant differences (*p* < 0.05) by using a Tukey test to compare in ANOVA.

As the four genes of *EoACP138*, *EoCYP316*, *EoCarE592* and *EoAchE989* were related to the detoxification ability of *E. oblique*, after the four genes of *EoACP138*, *EoCYP316*, *EoCarE592* and *EoAchE989* were silenced, the mortality of the fourth instar larvae after treatment with deltamethrin, fenpropathrin and chlorpyrifos was counted respectively to test whether the resistance of *E. oblique* larvae to pesticides was affected after the genes were silenced. The mortality of larvae after injection with dsRNAs targeting detoxifying genes was significantly greater than mortality in controls (CK and *GFP* dsRNA) and ranged from 44% to 59% when compared to the 12–19% mortality in larvae injected with CK and 25–33% mortality in larvae injected with *GFP* dsRNA (Figure 7).

## 4. Discussion

Pests cause significant crop losses worldwide. The use of broad-spectrum chemical pesticides to control pest damage is popular, but the following development of drug resistance has affected the sustainable use of pesticides. Therefore, it is very important for pest control to understand the expression levels of candidate genes potentially involved in insecticide resistance; however, with the rapid development of RNAi technology in insect gene function research and pest control [29], it has been found that the technology can be used to screen genes related to insecticide resistance and target genes for pest control [30]. 

In this study, firstly, the expression changes of genes of acid phosphatase *EoACP138*, cytochrome P450 *EoCYP316*, carboxylesterase *EoCarE592* and acetylcholinesterase *EoAchE989* were detected at 12 h, 24 h and 48 h under the stimulation of deltamethrin. The same trend of expression changes of *EoACP138* and *EoCarE592* genes reached the peak at 24 h, and then decreased; however, the changing trends of *EoCYP316* and *EoAchE989* are just the opposite. The expression of *EoCYP316* is the highest at 48 h, which is 15.73 times higher than that of the control group. *EoAchE989* is significantly increased at 12 h, and the expression is increased by 9.67 times. It is suggested that four genes may be involved in the detoxification process of *E. oblique*. It has been reported that arginine kinase TcAK1 in *Tribolium castaneum* significantly increased its mRNA level at 2 h and 4 h after deltamethrin stimulation, but recovered to the control level at 12 h, which indicated that different enzyme genes had different response mechanisms to deltamethrin stimulation [31].

Secondly, the expression levels of *EoACP138*, *EoCYP316*, *EoCarE592* and *EoAchE989* in the fourth instar larvae after 24 h treatment with chlorpyrifos and fenpropathrin were detected. The results showed that the expression levels of four genes were significantly increased at 24 h after treatment with chlorpyrifos and fenpropathrin, and the expression levels were increased by 5.14–6.74 times. The expression levels were increased by 8.23–14.64 times after treatment with fenpropathrin. It was speculated that the content of the above genes could be increased in 24 h after the treatment of pesticides in the worms to resist the pesticide damage. It has been reported that imidacloprid and β-cypermethrin could significantly induce the expression of RpCSP gene of *Rhopalosiphum padi*, suggesting that RpCSPs might be related to the response to exogenous toxic pesticides [32]. In this study, the expression levels of four genes (*EoACP138*, *EoCYP316*, *EoCarE592* and *EoAchE989*) were up-regulated after treatment with deltamethrin, chlorpyrifos and fenpropathrin, indicating that these four genes might be related to the detoxification metabolism of three pesticides by *E. oblique*.

RNAi is an indispensable means for gene function research in modern molecular biology experiments. At present, RNAi research on insects has involved gene function research on many common insects, such as *Spodoptera litura* [33], *Hyposidra talaca* [34], *Leptinotarsa decemlineata* [35], etc. The RNAi targeting *EoACP138*, *EoCYP316*, *EoCarE592* and *EoAchE989* resulted in significantly reduced expression of four target genes in *E*. *oblique*; this result was consistent with that observed in the diamondback moth, *plutella xylostella*, after oral administration of dsRNA for 24 h [36].

After injecting 200 ng of dsRNA, *E. oblique* were exposed to three pesticides, and a significant increase in larval mortality compared with the control was observed, indicating that RNAi increased the susceptibility of *E. oblique* to the three pesticides. The same phenomenon was also confirmed in *Nilaparvata lugens* [37]. In addition, after feeding Spodoptera litura dsRNA targeting SlGOBP2, reduced susceptibility to chrolorpyrifos was reported [38]. Similar results were observed in organophosphorus pesticide toxicity tests conducted by Meng et al. Following the injection of dsRNA of *Pp-AChE1* and *Pp-AChE2* encoded two acetylcholinesterase into the abdomen of *Pardosa pseudoannulata* [39]. This study demonstrated that the RNAi technology could be used to silence the detoxification-related genes to verify their function and improve the sensitivity of insects to pesticides, providing new ideas for the prevention and control of insects.

## 5. Conclusions

In this study, we observed that after pesticide stimulation, the expression levels of acidic phosphatase gene *EoACP138*, cytochrome P450 gene *EoCYP316*, carboxylesterase gene *EoCarE592*, and acetylcholinesterase gene *EoAchE989* were all significantly increased. After silencing the above genes by RNAi, it was found that the toxicity of the pesticide to *E. oblique* was significantly increased. The results indicated that the genes of *EoACP138*, *EoCYP316*, *EoCarE592* and *EoAchE989* were involved in metabolic detoxification pathways, which might be candidate genes for the biological control of *E. oblique* by RNAi. The results of this study will contribute to the implementation of more effective pest control strategies and the development of resistance to metabolic pesticides.

## Figures and Tables

**Table 1:** Sequences of the primers for quantitative real-time PCR.

Primer Name	Primer Sequence (5′-3′)
*Eo*RNA18S R	GAGAAACGGCTACCACATCCA
*Eo*RNA18S F	GCAAATGCTTTCGCTGATGTT
*EoACP138* R	TTCGCAGGGACAGTAGTGTAGGG
*EoACP138* F	ACATCCGCTCCACCGACTCTAC
*EoCYP316* R	CACCACCACCAACTTCTCACTCC
*EoCYP316* F	CGCAGGGTCGAGCAGCATATTAC
*EoCarE592* R	AGTGGCGAGAGGTAGTGGTAATGG
*EoCarE592* F	CGGCAACAACGGGCTGAAGG
*EoAchE989* R	ATCCATCAGCCTGTTGTCTGTTCG
*EoAchE989* F	GGAGCCCTTAACCGCCGAAAG

**Table 2:** Sequences of the primers for dsRNAs.

Primer Name	Primer Sequence (5′-3′)
*GFP* dsRNA R	taatacgactcactatagggGCTTCTCGTTCGGATCTTTG
*GFP* dsRNA F	taatacgactcactatagggGTGGAGTTGGACGGAGATGT
*EoACP138* dsRNA R	GATCACtaatacgactcactatagggGCCACGATGTTGAGGGTATC
*EoACP138* dsRNA F	GATCACtaatacactcactatagggCGATATTGATGCACCGTCAC
*EoCYP316* dsRNA R	GATCACtaatacgactcactatagggTGCTCGTTGTTTGAGAACCA
*EoCYP316* dsRNA F	GATCACtaatacgactcactatagggTCGGCTTTGGAAAAAGTGTT
*EoCarE592* dsRNA R	GATCACtaatacgactcactatagggGGAAAGACTCTTGCTGCCAC
*EoCarE592* dsRNA F	GATCACtaatacactcactatagggCAATGCGCAGATTGAGATGT
*EoAchE989* dsRNA R	GATCACtaatacgactcactatagggATTCGGGTGAATAGGCACAA
*EoAchE989* dsRNA F	GATCACtaatacgactcactatagggTTGGAATGTACGGCTTCCTC

The lowercase letters in the primers represent the sequence of the T7 promoter.

## Data Availability

Not applicable.
